# Drug survival and change of disease activity using a second janus kinase inhibitor in patients with difficult-to-treat rheumatoid arthritis who failed to a janus kinase inhibitor and subsequent biologics

**DOI:** 10.1186/s42358-024-00368-w

**Published:** 2024-04-15

**Authors:** Oh Chan Kwon, Wonho Choi, Soo Min Ahn, Ji Seon Oh, Seokchan Hong, Chang-Keun Lee, Bin Yoo, Min-Chan Park, Yong-Gil Kim

**Affiliations:** 1 Division of Rheumatology, Department of Internal Medicine, Gangnam Severance Hospital, Yonsei University College of Medicine, 211 Eonjuro, Gangnam- gu, 06273 Seoul, Korea; 2 Division of Rheumatology, Department of Internal Medicine, College of Medicine, University of Ulsan, Asan Medical Center, 88 Olympic-ro 43-gil, Songpa-gu, 05505 Seoul, Korea; 3 Department of Biomedical Informatics, Asan Medical Center, Seoul, Korea

**Keywords:** Rheumatoid arthritis, Janus kinase inhibitor, switching, Drug survival, Effectiveness

## Abstract

**Background:**

To assess the drug survival and change of disease activity using a second Janus kinase inhibitor (JAKi) after failure to a JAKi and subsequent biologic disease-modifying anti-rheumatic drugs (bDMARDs) in patients with difficult-to-treat rheumatoid arthritis (RA).

**Methods:**

This retrospective cohort study included 32 patients with difficult-to-treat RA who failed to a JAKi and subsequently to one or more bDMARDs and then switched to a second JAKi. To assess drug survival, electronic medical records of each patient were reviewed. Data on whether the second JAKi was discontinued, and the reasons for discontinuation were collected. The change of disease activity was assessed by analyzing changes in tender joint count (TJC), swollen joint count (SJC), patient’s global assessment of disease activity on a visual-analogue scale (VAS), erythrocyte sedimentation rate (ESR), C-reactive protein (CRP), Disease Activity Score for 28 joints with ESR (DAS28-ESR), and DAS28-CRP from baseline to that at six months from initiation of the second JAKi.

**Results:**

Overall, discontinuation of the second JAKi occurred in 20 (62.5%) patients. Primary failure, secondary failure, adverse events, and insurance coverage issues were the reasons for discontinuation in 9 (45.0%), 5 (25.0%), 2 (10.0%), and 4 (20.0%) patients, respectively. The estimated 2-year drug survival rate was 39.3%. In terms of change of disease activity, the second JAKi significantly improved TJC (*p* < 0.001), SJC (*p* < 0.001), VAS (*p* < 0.001), CRP (*p* = 0.026), DAS28-ESR (*p* < 0.001), and DAS28-CRP (*p* < 0.001) at 6-month compared with that at the baseline.

**Conclusions:**

Second JAKi could be a therapeutic option in patients with difficult-to-treat RA who have failed to a JAKi and subsequent bDMARDs.

## Background

Rheumatoid arthritis (RA) is an autoimmune disease characterized by synovitis leading to progressive joint damage [1]. Disease-modifying anti-rheumatic drugs (DMARDs), including conventional synthetic DMARDs (csDMARDs), biologic DMARDs (bDMARDs), and targeted synthetic DMARDs (tsDMARDs), are used for the treatment of RA [2, 3]. Of the DMARDs used in the treatment of RA, Janus kinase inhibitors (JAKis), categorized as tsDMARDs, are the most recently approved medication [4]. Clinical trials involving patients with RA have shown that JAKis with or without methotrexate have rapid and robust effects that are equivalent or even superior to that of bDMARDs [5–11]. Despite their efficacy, a fraction of patients with RA still fail to respond to JAKis and do not achieve low disease activity or remission [12, 13]. The European League Against Rheumatism (EULAR) recommendation states that, switching to a bDMARD or another tsDMARD in these patients should be considered [2]. However, there are limited data about the use of other JAKis after failure to respond to one JAKi. Only one recent retrospective study has shown that switching from one JAKi to another sequentially could be safe and effective [14]. However, as only patients with back to back use of different JAKis were included in that study [14], a clinical question remains as to whether the observed effectiveness of the second JAKi after failure to a first JAKi still applies to patients with difficult-to-treat RA who failed to a JAKi and subsequently to one or more bDMARDs.

Here, we aimed to evaluate the drug survival and change of disease activity using a second JAKi, in patients with difficult-to-treat RA who failed to a JAKi and subsequent bDMARDs.

## Methods

### Patients

Patients with RA who failed to a JAKi and have been switched to one or more bDMARDs and then to another JAKi at two tertiary referral hospitals in Seoul, South Korea, between August 2015 and June 2021 were included. All patients included fulfilled the 2010 American College of Rheumatology/EULAR classification criteria for RA [15]. At the time of initiation of the second JAKi, all patients met the definition of “difficult-to-treat RA” [16] which includes: (i) failure to ≥ 2 b/tsDMARDs with different mechanisms of action after failure to csDMARDs, (ii) signs suggestive of active/progressive disease (e.g. Disease Activity Score for 28 joints [DAS28] > 3.2), and (iii) management of signs and/or symptoms perceived as problematic by the rheumatologist and/or the patient. Usual care was applied to the patients. All JAKis were used in full doses: tofacitinib, 5 mg twice a day; baricitinib, 4 mg once a day; and upadacitinib, 15 mg once a day. In all cases, the switch from bDMARD to JAKi was made directly without washout period. Patients were retrospectively reviewed from the date of initiation of the second JAKi to the date of discontinuation of that JAKi, or December 31, 2021, or the last follow-up date, whichever came first. This study was approved by the Institutional Review Board (IRB) of Gangnam Severance Hospital (IRB No: 3-2021-0446) and Asan Medical Center in Seoul, South Korea (IRB No: 2014 − 0237). Requirement of informed consent was waived due to the retrospective nature of the study.

### Outcomes

The outcome of primary interest was drug survival of the second JAKi. To assess drug survival of the second JAKi, the electronic medical records of each patient were retrospectively reviewed and whether that JAKi was discontinued was determined. Patients who discontinued their JAKis owing to primary failure, secondary failure, adverse events, and insurance coverage issues were detected. Primary failure was defined as DAS28 with erythrocyte sedimentation rate (DAS28-ESR) ≤ 3.2 not achieved atsix months of treatment. Secondary failure was defined as loss of this condition thereafter. We also assessed change of disease activity between baseline and 6-month as a secondary outcome. Change of disease activity was assessed by change from baseline to that at six months from the initiation of the second JAKi in the following covariates: tender joint count (TJC), swollen joint count (SJC), patient’s global assessment of disease activity on a visual-analogue scale (VAS) ranging from 0 to 100, ESR, C-reactive protein (CRP), DAS28-ESR, and DAS28-CRP.

### Covariates

The following covariates at the time of initiation of the second JAKi were collected: age, sex, disease duration, positivity for rheumatoid factor and anti-cyclic citrullinated peptide antibody, erosion on x-ray, number of bDMARDs used prior to the first JAKi, the first JAKi used, duration of exposure to the first JAKi, reason for discontinuation of the first JAKi, number of bDMARDs used between JAKis, time interval between the use of JAKis, the second JAKi that was initiated, and the use of methotrexate and glucocorticoid.

### Statistical analysis

The sample size needed to achieve a power of 80% and a significance level of 5% (two-sided) for detecting an effect size of 0.6 on disease activity indices (TJC, SJC, VAS, ESR, CRP, DAS28-ESR, and DAS28-CRP) between pairs (baseline and 6-month assessment) was calculated. The effect size refers to the mean of the paired differences divided by the standard deviation of paired differences. The calculated sample size was 25 pairs. Continuous variables were represented by their median value (interquartile range [IQR]), and categorical variables were represented as numbers (%). Kaplan-Meier analysis was conducted to visualize drug survival of the second JAKi. Wilcoxon signed rank test was used to compare the differences in TJC, SJC, VAS, ESR, CRP, DAS28-ESR, and DAS28-CRP at baseline and at six months of the second JAKi treatment. Patients who discontinued the JAKi before 6-month owing to adverse events or insurance coverage issues were excluded in this analysis. A p value of < 0.05 was considered statistically significant. All analyses were conducted using the SPSS software (version 25.0; IBM Corporation, Armonk, NY, USA), and graphical representations were generated using GraphPad Prism (version 7.0; GraphPad Software Inc., San Diego, CA, USA).

## Results

### Patient characteristics

A total of 32 patients with RA who failed to a JAKi and were switched to one or more bDMARDs and then to another JAKi were included. The median age of the patients was 56.5 (IQR: 48.3, 61.0) years, and 93.8% of the patients were female. Tofacitinib and baricitinib were the JAKis previously used in 30 (93.8%) and 2 (6.3%) patients, respectively. The median number of bDMARDs used between the first and second JAKi was 1.0 (IQR: 1.0, 2.0). Tofacitinib, baricitinib, and upadacitinib were the second JAKi in 2 (6.3%), 29 (90.6%), and 1 (3.1%) patient, respectively. During the use of the second JAKi, methotrexate was concomitantly used in 22 (68.8%) patients. In these 22 patients, methotrexate was used at a stable dose with a median dose of 10.0 (9.4–13.1) mg/week. Detailed characteristics of the patients at the initiation of the second JAKi are summarized in Table 1.

**Table 1:** Patient characteristics at the initiation of second JAK inhibitor

Characteristics	*N* = 32
Age, years, median (IQR)	56.5 (48.3, 61.0)
Female, n (%)	30 (93.8)
Disease duration, years, median (IQR)	13.5 (8.4, 21.5)
RF positive, n (%)	26 (81.3)
Anti-CCP Ab positive, n (%)^a^	23 (85.2)
Erosion on x-ray, n (%)	26 (81.3)
Number of bDMARDs prior to the first JAKi, median (IQR)	1.0 (0.0, 2.0)
First JAKi, n (%)	
Tofacitinib	30 (93.8)
Baricitinib	2 (6.3)
Duration of first JAKi exposure, months, median (IQR)	8.0 (3.5, 17.0)
Reason for discontinuation of first JAKi, n (%)	
Primary failure	16 (50.0)
Secondary failure	6 (18.8)
Adverse events	10 (31.3)
Number of bDMARDs between JAKis, median (IQR)	1.0 (1.0, 2.0)
Interval between JAKis, months, median (IQR)	10.0 (3.0, 22.0)
Second JAKi, n (%)	
Tofacitinib	2 (6.3)
Baricitinib	29 (90.6)
Upadacitinib	1 (3.1)
Concomitant medication	
Methotrexate, n (%)	22 (68.8)
Methotrexate dose, mg/week, median (IQR)	10.0 (9.4–13.1)
Glucocorticoid, mg/day^b^, median (IQR)	5.0 (0.0, 5.0)

^a^Patients (*n* = 5) with missing data excluded

^b^mg/day of prednisolone or its equivalent

JAKi, Janus kinase inhibitor; RF, Rheumatoid factor; Anti-CCP Ab, Anti-cyclic citrullinated peptide antibody; bDMARDs, biologic disease-modifying anti-rheumatic drugs; TJC, Tender joint count; SJC, Swollen joint count; VAS, Visual analogue scale; ESR, Erythrocyte sedimentation rate; CRP, C-reactive protein; DAS28-ESR, Disease activity score 28 - erythrocyte sedimentation rate

### Drug survival

**Fig. 1:**
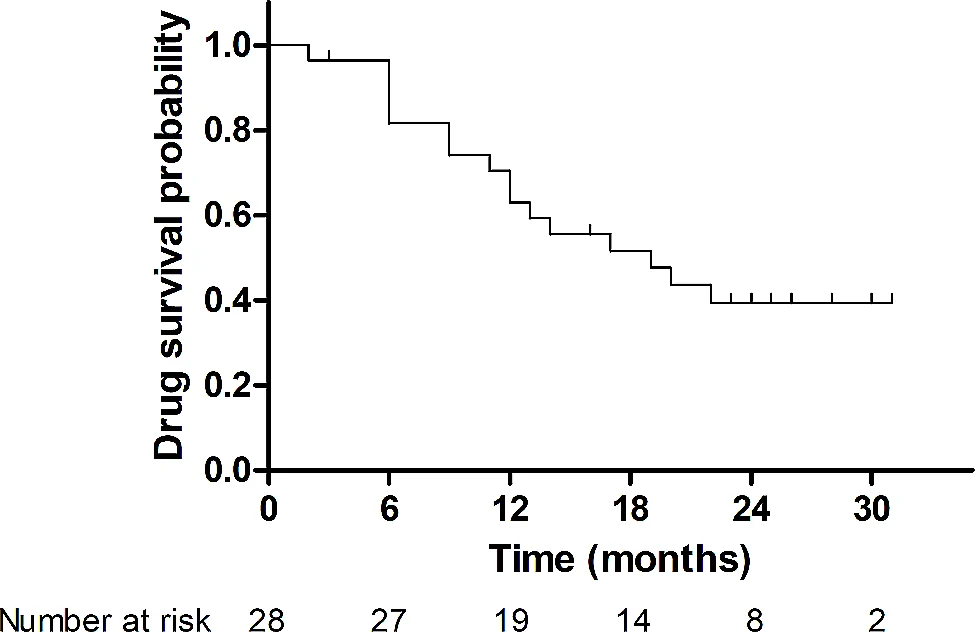
Drug survival rate of another JAK inhibitor after failure to a JAKi and subsequent bDMARD(s). JAK, Janus kinase; bDMARD, biologic disease-modifying anti-rheumatic drug

The median observation duration (i.e. duration of exposure to the second JAKi) was 13.5 (IQR: 6.3, 23.8) months. Overall, 20 patients (62.5%) discontinued treatment with the second JAKi. The median time of discontinuation of the second JAKi was after 9.0 (IQR: 4.5, 13.8) months of treatment. Among the 20 patients who discontinued the second JAKi, primary failure, secondary failure, adverse events, and insurance coverage issues were the reasons for the discontinuation in 9 (45.0%), 5 (25.0%), 2 (10.0%), and 4 (20.0%) patients, respectively (Table 2). Excluding the 4 patients who discontinued the JAKi due to insurance coverage issue, a total of 4 patients discontinued the JAKi within 6-month of treatment. Of the 4 patients, 3 patients discontinued due to primary failure, and 1 patient discontinued due to adverse event (severe nausea). When patients who discontinued the second JAKi due to insurance coverage issues were excluded, the estimated drug survival rates at 6-month, 1-year, and 2-year were 85.7%, 70.5%, and 39.3%, respectively (Fig. 1).

**Table 2:** Drug survival of the second JAK inhibitor

	*N* = 32
Duration of the second JAKi exposure, months, median (IQR)	13.5 (4.5, 23.8)
Discontinuation of the second JAKi, n (%)	20 (62.5)
Time to discontinuation of the second JAKi, months, median (IQR)	9.0 (3.3, 13.8)
Reasons for discontinuation of the second JAKi, n (%)	
Primary failure	9 (45.0)
Secondary failure	5 (25.0)
Adverse events	2 (10.0)
Insurance coverage issues	4 (20.0)

JAKi, Janus kinase inhibitor

### Change of disease activity

**Fig. 2:**
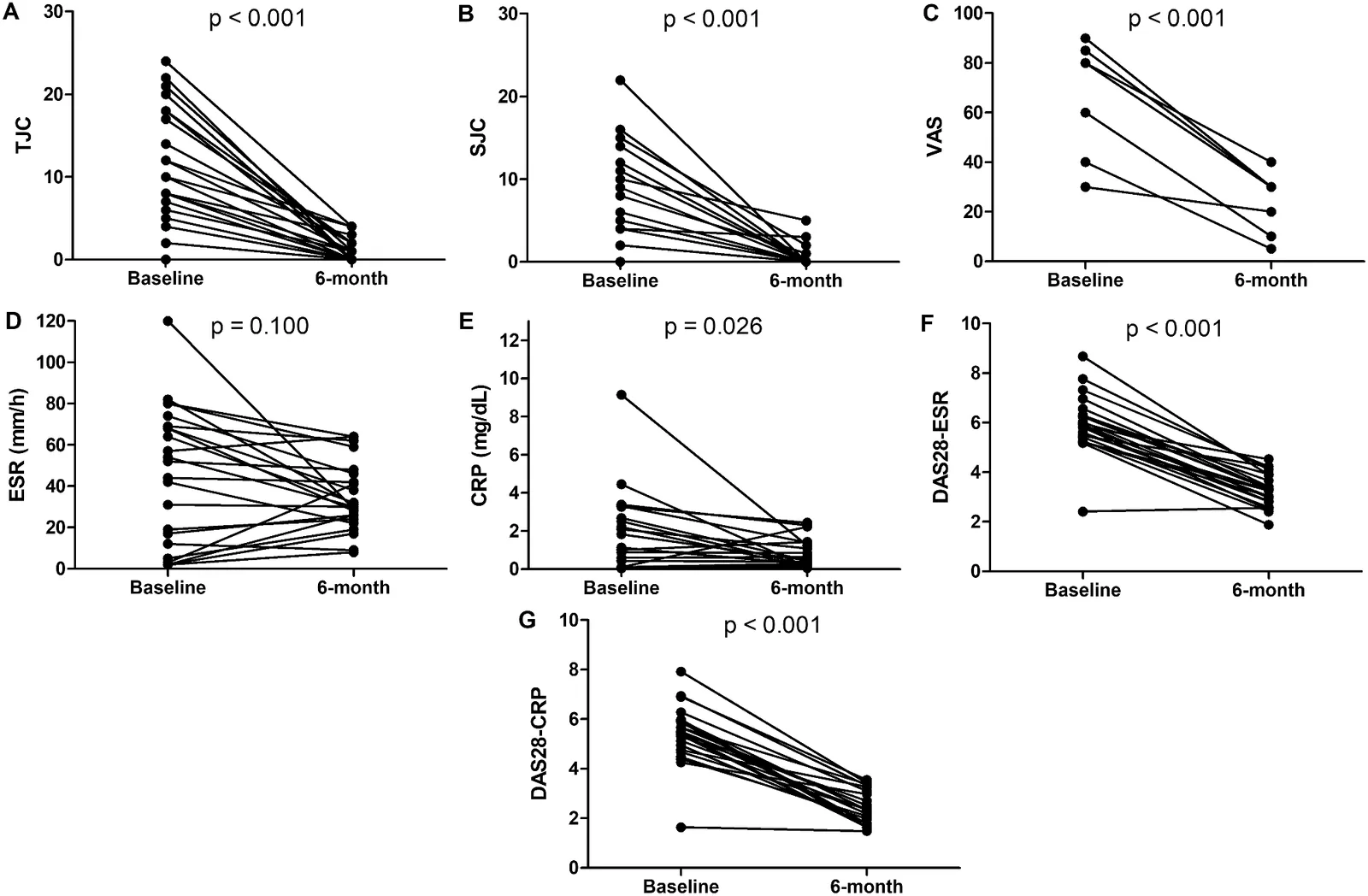
Changes in disease activity parameters between baseline and at six months from the initiation of a second JAK inhibitor. (**A**) TJC, (**B**) SJC, (**C**) VAS, (**D**) ESR, (**E**) CRP, (**F**) DAS28-ESR, and (**G**) DAS28-CRP. JAK, Janus kinase; TJC, Tender joint count; SJC, Swollen joint count; VAS, Visual analogue scale; ESR, Erythrocyte sedimentation rate; CRP, C-reactive protein; DAS28, Disease activity score

Change of disease activity using the second JAKi at six months of treatment is shown in Table 3; Fig. 2. Compared with the baseline, TJC (10.0 [IQR: 6.0, 18.0] vs. 0.0 [IQR: 0.0, 2.0], *p* < 0.001), SJC (6.0 [IQR: 4.0, 12.0] vs. 0.0 [IQR: 0.0, 0.0], *p* < 0.001), VAS (85.0 [IQR: 80.0, 90.0] vs. 30.0 [IQR: 30.0, 30.0], *p* < 0.001), CRP (1.01 [IQR: 0.10, 2.68] mg/dL vs. 0.38 [IQR: 0.13, 1.23] mg/dL, *p* = 0.026), DAS28-ESR (5.84 [IQR: 5.41, 6.30] vs. 3.28 [IQR: 2.81, 3.89], *p* < 0.001), and DAS28-CRP (5.48 [IQR: 4.75, 5.94] vs. 2.34 [IQR: 1.78, 3.10], *p* < 0.001) were significantly lower at six months of treatment with the second JAKi. On the other hand, ESR (52.0 [IQR: 12.0, 69.0] mm/h vs. 30.0 [IQR: 24.0, 46.0] mm/h, *p* = 0.100) at baseline and at six months of the second JAKi treatment did not differ significantly.

**Table 3:** Change of disease activity using the second JAK inhibitor at 6-month

	Baseline	6-month	*P* value^a^
Number of patients at assessment, n (%)	32 (100.0)	27 (84.4)	N/A
TJC	10.0 (6.0, 18.0)	0.0 (0.0, 2.0)	< 0.001
SJC	6.0 (4.0, 12.0)	0.0 (0.0, 0.0)	< 0.001
VAS	85.0 (80.0, 90.0)	30.0 (30.0, 30.0)	< 0.001
ESR, mm/h	52.0 (12.0, 69.0)	30.0 (24.0, 46.0)	0.100
CRP, mg/dL	1.01 (0.10, 2.68)	0.38 (0.13, 1.23)	0.026
DAS28-ESR	5.84 (5.41, 6.30)	3.28 (2.81, 3.89)	< 0.001
DAS28-CRP	5.48 (4.75, 5.94)	2.34 (1.78, 3.10)	< 0.001

^a^The P value of Wilcoxon signed rank test, which included 27 patients who had their disease activity assessed at both baseline and 6-month

JAK, Janus kinase; TJC, Tender joint count; SJC, Swollen joint count; VAS, Visual analogue scale; ESR, Erythrocyte sedimentation rate; CRP, C-reactive protein; DAS28, Disease activity score 28

## Discussion

In this study, we showed that in patients with difficult-to-treat RA who failed to bDMARDs after failure to a JAKi, a second JAKi has a drug survival rate of up to 40% at 2-year. Given that all patients included had difficult-to-treat RA, the drug survival rate was acceptable, suggesting that the second JAKi is a potential therapeutic option available to patients with difficult-to-treat RA who failed to a JAKi and one or more subsequent bDMARDs.

In vitro kinase assays have demonstrated that different JAKis have different JAK selectivity. Tofacitinib is a potent inhibitor of JAK1 and JAK3 and is less specific to JAK2 and tyrosine kinase 2 (TYK2); baricitinib is a selective JAK1 and JAK2 inhibitor with moderate activity against TYK2 and less activity against JAK3; and upadacitinib is a selective JAK1 inhibitor [17–19]. Considering that distinct cytokine signaling pathways are mediated by varying JAK complexes, different JAKis with different JAK selectivity, may have different effects in the treatment of RA [20]. This could be a rationale for using another JAKi in patients with RA who have previously failed to a JAKi. Indeed, a previous study has reported sequential use of second JAKi after failure to a first JAKi as an efficacious option in patients with RA [14]. Our data add to the previous study that a second JAKi could also be considered as a therapeutic option when a patient with RA has failed to a JAKi and to subsequent bDMARDs.

Regarding drug survival, the estimated 2-year drug survival rate of the second JAKi was 39.3%. This drug survival rate is relatively low compared with a previous post hoc analysis evaluating drug survival of JAKi (tofacitinib, in particular) in patients with RA that reported an estimated 2-year drug survival rate of 75.5% [21]. This is not surprising considering that all patients included in our study fall into the definition of difficult-to-treat RA [16] at the time of initiation of the second JAKi. As we included only the patients with RA who failed to a JAKi and one or more subsequent bDMARDs, all patients received at least two ts/bDMARDs prior to the second JAKi. In contrast, the mean number of bDMARDs used by the patients included in the previous post hoc analysis prior to the use of tofacitinib was 1.6 [21]. Therefore, although the drug retention rate of the second JAKi in our study seems relatively low, it could be interpreted that as much as 40% of patients with difficult-to-treat RA who previously failed to a JAKi still have chance to improve upon treatment with another JAKi. Moreover, in our country, as per the national insurance reimbursement policy, patients with RA using JAKi or bDMARD are mandated to get their disease activity assessed every 6-month. Only if the effectiveness observed at the first 6-month of use is maintained thereafter can the JAKi or bDMARDs be used persistently. Given these circumstances, we presume that drug survival could be considered as a proxy of drug effectiveness in our study population.

The use of the second JAKi was effective at 6-month in terms of improving TJC, SJC, VAS, CRP, and DAS28. Given that all patients included had difficult-to-treat RA, the effect of the second JAKi on the change of disease activity observed at 6-month is encouraging. However, as shown in Fig. 2, the degree of change in disease activity parameters varied among patients. For instance, ΔDAS28-ESR (i.e. change of DAS28-ESR from baseline to at six months of use of the second JAKi) was as large as -4.79 in one patient, whereas in another patient DAS28-ESR was rather higher at six months of treatment (ΔDAS28-ESR = 0.15). An important clinical question arises as to whether there is a subgroup of patients that responds better than the others. In particular, given that all patients failed to a JAKi previously, we sought whether the reason for discontinuation of the previous JAKi is associated with the effectiveness or drug survival of the second JAKi. When ΔDAS28-ESR was compared according to the reasons for discontinuation of the previous JAKi, no significant difference was observed among groups (*p* = 0.486, data not shown in the Results). In addition, the drug survival rate of the second JAKi also did not differ among patients who discontinued treatment with the previous JAKi for different reasons (*p* = 0.423, data not shown in the Results). Moreover, as shown in Table 4, no association was observed between the reason for discontinuation of the previous JAKi and the outcome of the second JAKi, indicating that the reason for the discontinuation of the previous JAKi does not necessarily mean that the second JAKi will be discontinued for the same reason. For instance, of the 16 patients who discontinued treatment with the previous JAKi owing to primary failure, excluding one patient who discontinued the second JAKi of insurance coverage issue, four patients experienced primary failure with the second JAKi, three patients experienced secondary failure with the second JAKi, one patient experienced adverse event with the second JAKi, and the other seven patients were maintained on the second JAKi as it was effective (Table 4). Therefore, the so-called class effect does not seem to apply to JAKis, and switching to another JAKi after failure to a JAKi and subsequent bDMARDs, could be considered regardless of the reasons for the discontinuation of the previous JAKi.

**Table 4:** Reasons for discontinuation of first and second JAK inhibitors

Patients (Age/Sex)	First JAKi		Second JAKi	
	Drug	Reasons for discontinuation	Drug	Reasons for discontinuation
#1 (63/F)	Tofacitinib	Primary failure	Baricitinib	Secondary failure
#2 (62/F)	Tofacitinib	Primary failure	Baricitinib	Secondary failure
#3 (48/F)	Tofacitinib	Primary failure	Baricitinib	Adverse event
#4 (45/F)	Tofacitinib	Primary failure	Upadacitinib	N/A^a^
#5 (40/F)	Tofacitinib	Secondary failure	Baricitinib	Primary failure
#6 (61/F)	Tofacitinib	Secondary failure	Baricitinib	Primary failure
#7 (58/F)	Tofacitinib	Primary failure	Baricitinib	Primary failure
#8 (66/M)	Tofacitinib	Secondary failure	Baricitinib	Secondary failure
#9 (49/F)	Tofacitinib	Primary failure	Baricitinib	Primary failure
#10 (56/F)	Tofacitinib	Primary failure	Baricitinib	Primary failure
#11 (59/F)	Tofacitinib	Primary failure	Baricitinib	Primary failure
#12 (60/M)	Tofacitinib	Primary failure	Baricitinib	Secondary failure
#13 (68/F)	Tofacitinib	Secondary failure	Baricitinib	Adverse event
#14 (55/F)	Tofacitinib	Secondary failure	Baricitinib	Insurance coverage issue
#15 (35/F)	Tofacitinib	Primary failure	Baricitinib	Insurance coverage issue
#16 (50/F)	Tofacitinib	Primary failure	Baricitinib	N/A^a^
#17 (49/F)	Tofacitinib	Primary failure	Baricitinib	N/A^a^
#18 (49/F)	Tofacitinib	Primary failure	Baricitinib	N/A^a^
#19 (58/F)	Tofacitinib	Primary failure	Baricitinib	N/A^a^
#20 (35/F)	Tofacitinib	Secondary failure	Baricitinib	N/A^a^
#21 (58/F)	Tofacitinib	Primary failure	Baricitinib	N/A^a^
#22 (65/F)	Tofacitinib	Primary failure	Baricitinib	N/A^a^
#23 (40/F)	Tofacitinib	Adverse event	Baricitinib	Primary failure
#24 (57/F)	Tofacitinib	Adverse event	Baricitinib	Secondary failure
#25 (56/F)	Tofacitinib	Adverse event	Baricitinib	Primary failure
#26 (35/F)	Tofacitinib	Adverse event	Baricitinib	Primary failure
#27 (68/F)	Tofacitinib	Adverse event	Baricitinib	Insurance coverage issue
#28 (34/F)	Baricitinib	Adverse event	Tofacitinib	Insurance coverage issue
#29 (54/F)	Tofacitinib	Adverse event	Baricitinib	N/A^a^
#30 (57/F)	Tofacitinib	Adverse event	Baricitinib	N/A^a^
#31 (62/F)	Tofacitinib	Adverse event	Baricitinib	N/A^a^
#32 (61/F)	Baricitinib	Adverse event	Tofacitinib	N/A^a^

^a^Not applicable because the JAKi was continued

JAKi, Janus kinase inhibitor

The following limitations should be noted in our study. First, we lack data on other disease activity parameters including Clinical Disease Activity Index and Simplified Disease Activity Index. Second, the observation duration was relatively short and the number of patients small to make a robust conclusion. Third, this study was retrospective in design. As this was a retrospective study, glucocorticoid was not used in a predefined standardized protocol. Fourth, as this was a study based on an electronic medical record review, information on adverse events and treatment discontinuation may have been underreported. Further prospective randomized controlled trials with larger sample size and longer follow-up are needed to confirm our observations.

## Conclusions

In summary, we found that switching to another JAKi in patients with difficult-to-treat RA who failed to a JAKi and subsequent bDMARDs has an estimated 2-year drug survival rate of approximately 40%, which is acceptable given the refractoriness of the study population. Furthermore, the second JAKi was effective in improving disease activity at six months of treatment. Therefore, another JAKi could be considered as a therapeutic option when patients with difficult-to-treat RA have failed to a JAKi and subsequent bDMARDs.

## Data Availability

All data generated or analyzed during this study are included in this article.

## Abbreviations

RA: rheumatoid arthritis
DMARDs: disease-modifying anti-rheumatic drugs
bDMARDs: biologic disease-modifying anti-rheumatic drugs
tsDMARDs: targeted synthetic disease-modifying anti-rheumatic drugs
JAKis: janus kinase inhibitors
EULAR: European League Against Rheumatism
DAS28: Disease Activity Score for 28 joints
IRB: Institutional Review Board
DAS28-ESR: Disease Activity Score for 28 joints with erythrocyte sedimentation rate
TJC: tender joint count
SJC: swollen joint count
VAS: visual-analogue scale
CRP: C-reactive protein
IQR: interquartile range
TYK2: tyrosine kinase 2
